# A solid-phase extraction method using Transcarpathian clinoptilolite for preconcentration of trace amounts of terbium in water samples

**DOI:** 10.1186/s13065-015-0118-z

**Published:** 2015-08-27

**Authors:** Volodymyr O. Vasylechko, Galyna V. Gryshchouk, Victor P. Zakordonskiy, Olga Vyviurska, Andriy V. Pashuk

**Affiliations:** Faculty of Chemistry, Ivan Franko National University of Lviv, 6 Kyryla and Mefodiya St., 79005 Lviv, Ukraine; Department of Chemistry and Physics, Lviv Academy of Commerce, 9 Samchuka St., 79011 Lviv, Ukraine; Department of Environmental Safety and Nature Protection, Lviv Polytechnic National University, 12 Bandera St., 79013 Lviv, Ukraine

**Keywords:** Preconcentration, Terbium, Solid-phase extraction, Clinoptilolite

## Abstract

**Background:**

In spite of the fact that terbium is one of the rarest elements in the Earth’s crust, it is frequently used for the production of high technological materials. At the result, an effective combination of sample preparation procedure and detection method for terbium ions in different matrices is highly required. The solid-phase extraction procedure with natural Transcarpathian clinoptilolite thermally activated at 350 °C was used to preconcentrate trace amounts of terbium ions in aqueous solutions for a final spectrophotometric determination with arsenazo III.

**Results:**

Thermogravimetric investigations confirmed the existence of relations between changes that appeared during dehydratation of calcined zeolite and its sorption affinity. Since the maximum of sorption capacity towards terbium was observed at pH 8.25, a borate buffer medium (2.5 · 10^−4^ М) was used to maintain ionic force and solution acidity. Terbium was quantitatively removed from the solid-phase extraction column with a 1.0 M solution of sodium chloride (pH 2.5). The linearity of the proposed method was evaluated in the range of 2.5-200 ng · mL^−1^ with detection limit 0.75 ng · mL^−1^.

**Conclusions:**

Due to acceptable recoveries (93.3–102.0 %) and RSD values (6–7.1) from spiked tap water, the developed method can be successfully applied for the determination of trace amounts of terbium ions in the presence of major components of water.

**Graphical abstract:**
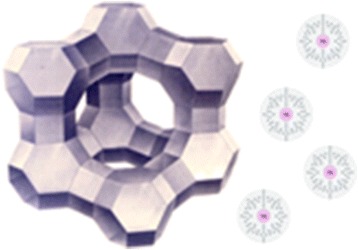
Sorption of terbium(III) ions on clinoptilolite

## Background

Terbium belongs to the rarest elements, participating with 1.1 · 10^−4^ % in the composition of the Earth’s crust [1], and this is only 1.0 % among all lanthanides including yttrium. At the same time terbium compounds have been widely applied for luminophores, magnetic and laser materials. Furthermore, this lanthanide was detected in sea and mineral waters [2] as well as in some wines at the microlevel. For example, the content of terbium and other rare earth elements was used for authentication of Hungarian wines [3]. In general, the determination of microelements from real samples and wastewaters requires a proper sample preparation procedure, including such steps as preconcentration, separation and isolation from natural objects and process liquors. In this case, ion exchange and extraction chromatography were found to be effective and fast for the separation of trace amounts of rare earth elements [4, 5]. At the same time, the solid-phase extraction (SPE) has also become quite popular sample preparation procedure for trace analysis [5–9], allows to reduce solvent consumption due to a simplified procedure of the solvent removal. Furthermore, SPE could be easily combined with the selective and high sensitive methods of the analysis, e.g. atomic absorption spectroscopy [9, 10] and inductively coupled plasma spectroscopy [5–7]. A variety of sorbents such as modified high dispersed silica [5, 11, 12], activated carbon [5, 13, 14], polyurethane foams [15, 16], polymeric resins [9, 17], carbosilicon [18], synthetic zeolites [19] are commonly used for solid phase extraction. In recent years, the popularity of natural zeolites has increased for SPE applications [6, 10, 20–29], because of a number of advantages over the other sorbents. For example, these natural aluminosilicate minerals contain pores and cavities with strictly defined size and shape, and it provides very effective concentration and separation of organic and inorganic compounds. In addition, zeolites have mechanical strength, good stability in aggressive medium and under thermal treatment, ability to sorb the trace amounts of analytes, high sorption capacity and selectivity, possibility of easy modification and regeneration of the sorbent, low cost and accessibility. The sorption properties of Transcarpathian clinoptilolite towards terbium ions were described in our previous work [30]. The aim of this study is to complete these investigations and to develop a simple sample preparation procedure for the spectrophotometric detection of trace amounts of terbium ions in aqueous solutions.

## Experimental

### Reagents and apparatus

All used reagents were of analytical grade. Standard aqueous solutions of terbium(III) nitrate (1.0 mg · mL^−1^) were prepared by dissolution of metallic terbium (99.9 % purity) in nitric acid (1:1). The following solutions were also used for the experiments: a 0.05 % solution of sulfarsazene in a 0.05 M borax solution, a 0.05 % aqueous solution of arsenazo III, a 1 % ascorbic acid solution, a formic buffer solution (60 mL of formic acid and 28 g of NaOH are dissolved in 1.0 L of water), a 1 M sodium chloride solution, a 5 % sulphosalicylic acid solution, a 0.2 M EDTA solution, a 0.1 M aqueous solutions of NaOH and Na_2_B_4_O_7_, and a borate buffer solution with pH 8.25 (595 mL of 0.05 M Na_2_B_4_O_7_ diluted to 1.0 L with 0.1 M HCl). Clinoptilolite samples with 85–90 % of the main component content were taken from the deposit near the village Sokirnytsia in the Ukrainian Transcarpathian region. The specific surface area measured with water sorption was found to be 59 m^2^ · g^−1^ [31]. The chemical composition of Transcarpathian clinoptilolite is (in %): SiO_2_, 67.29; TiO_2_, 0.26; Al_2_O_3_, 12.32; Fe_2_O_3_, 1.26; FeO, 0.25; MgO, 0.99; CaO, 3.01; Na_2_O, 0.66; K_2_O, 2.76; H_2_O, 10.90 [32]. The thermal heating of Transcarpathian clinoptilolite was carried out for 2.5 h in the oven. The results of the X-ray analysis of the calcined Transcarpathian clinoptilolite were described in details in our previous study [33]. Spectrophotometric determination was performed on a HACH DR/4000 V spectrophotometer. X-ray fluorescence investigations were carried out on an Expert 3 L multi element rapid response analyzer (INAM, Ukraine) with semiconducting PIN-detector (AMPTEK, USA) on thermoelectric cooling. A low-power X-ray tube was operated at 45 kV (current 0.1 mA, output 4.5 W). In this case, 2 mL of the sample was placed into the cuvette, and Tb Lα line radiation was measured for 285 s. The Paulik–Paulik–Erdey Q–1500D (MOM, Hungary) system was employed for thermogravimetric analysis.

### Adsorption measurements

The sorption properties of clinoptilolite were studied under dynamic conditions using a peristaltic pump, and the procedure was described in details in our previous paper [34]. The metal solutions were passed through a sorption cartridge filled with 0.6 g of sorbent at a flow rate of 3 mL · min^−1^. A passing moment of terbium(III) ions (LDL, 100 ng · mL^−1^) was detected visually and/or spectrophotometrically at 540 nm with sulfarsazene as a chromogenic reagent, and additionally confirmed by X-ray fluorescence.

An acidified solution of sodium sulphate and mineral acids solutions were used for desorption of Tb(III) ions from clinoptilolite. In this case 15 mL of eluent was passed through the sorption cartridge at a flow rate of 1 mL · min^−1^. The obtained eluate was collected into a volumetric glass flask and made up to 25 mL using double-distilled water.Since selectivity of determination of Tb(III) ions with sulfarsazene was insufficient for the analysis of desorption filtrates, a content of Tb(III) ions was found spectrophotometrically with arsenazo III as a reagent [35, 36]. Interference of Fe(III), Al(III), Ca(II) and Mn(II) ions was eliminated by the addition of ascorbic and sulphosalicylic acids, EDTA and Seignette salt to the system. The adsorption and desorption studies were carried out at a temperature of 20 ± 1 °C.

### Method of spectrophotometric determination with arsenazo III

5 mL of an 1 % ascorbic acid solution, 4 mL of a 4 % sulphosalicylic acid solution, 5 mL of a 0.2 M EDTA solution, 3 mL of a 5 % Seignette salt solution were mixed with 25 mL of the analysed solution (pH ~ 1). After 2 min, a mixture of 1 mL of a formic buffer solution (pH 3.5) and 4 mL of a 0.05 % arsenazo III solution was diluted with double-distilled water to approximately 40 mL and regulated to pH 2.6 ± 0.1 with a 0.1 M NaOH. The final volume of the solution was made up to 50 mL with double-distilled water, and the absorbance was measured spectophotometrically at 650 nm against the blank solution which contains all the reagents except for terbium(III) ions. The quantitative determination of terbium(III) ions was carried out in the final volume of the solution (in concentration range of 0.1–2 μg · mL^−1^).

### Thermogravimetric analysis

The analysis was performed under air conditions with the heating rate 10 °C · min^−1^ from 50 to 900 °C with Al_2_O_3_ as a standard material. Clinoptilolite samples (grain size 0.20–0.31 mm) weighting 500–510 mg were placed in corundum crucibles. The sorbent was preliminary heated in a drying oven or a muffle oven during 2.5 h and stored in a desiccator above a saturated solution of H_2_SO_4_.

## Results and discussion

**Fig. 1:**
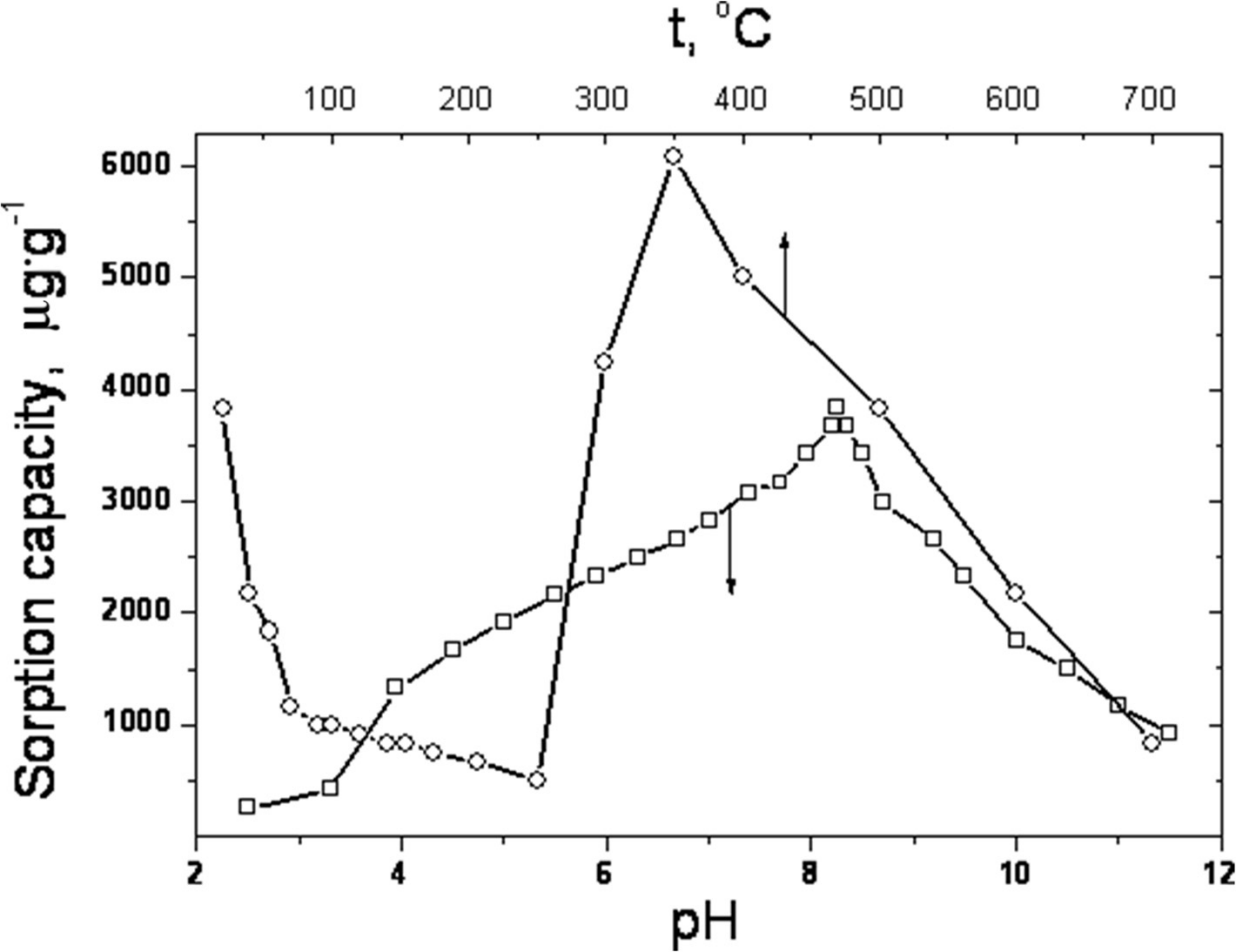
Dependence of clinoptilolite sorption capacity of terbium(III) ions on a pH value of the aqueous solution and thermal treatment carried out in the range from 20 to 700 °C (pH 8.25) (concentration of Tb(III) – 1 μg•mL^−1^; pH 8.25; a flow rate −3 mL•min^−1^; time of heat treatment – 2.5 h)

As can be seen from the Fig. 1, the sorption capacity of Transcarpathian clinoptilolite towards terbium(III) ions considerably depends on the pH of analyte solution and previous thermal treatment of the used zeolite. The most effective sorption was observed in the weak alkaline solutions (pH 8.25), where according to our previous studies [30], terbium(III) ions exist in three cation forms of Tb^3+^ (~25 %), TbOH^2+^ (~50 %) and Tb(OH)_2_^+^ (~25 %). In order to maintain pH and improve the accuracy of the investigations, a borate buffer was added to terbium solution. Consequently, it was found that the same amounts of terbium(III) ions were concentrated from the solution adjusted to pH 8.25 with sodium hydroxide at once and from the solution which was firstly neutralized, then buffered to pH 8.25. Moreover, the use of the buffer solution provides a possibility to keep constant ionic strenght, and minimize the negative influence of different admixtures on the effectiveness of terbium preconcentration.

As to the thermal treatment of Transcarpathian clinoptilolite, a minimum at 250 °C and a maximum at 350 °C of sorption capacity for terbium were observed over a narrow temperature range (Fig. 1). These observations confirmed the connection between zeolite structural changes during dehydration processes and its sorption abilities [23–31, 33, 34, 37]. At the same time, only partial rehydration of zeolite could be observed in distilled water, e.g. water content of Transcarpathian clinoptilolite was reduced by 18 wt. % after thermal treatment at 500 °C. As a result, the sorption effectiveness of thermally activated zeolite towards trace element ions was not further diminished in aqueous solutions.

**Fig. 2:**
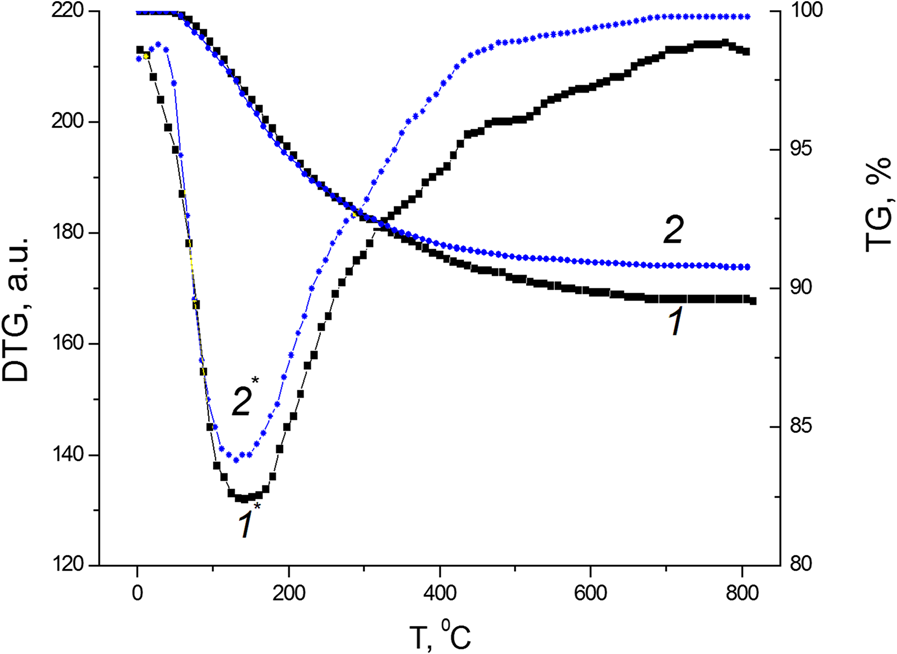
TG and DTG-curves of natural clinoptilolite (1, 1*) and clinoptilolite heated at 500 °С (2, 2*)

**Fig. 3:**
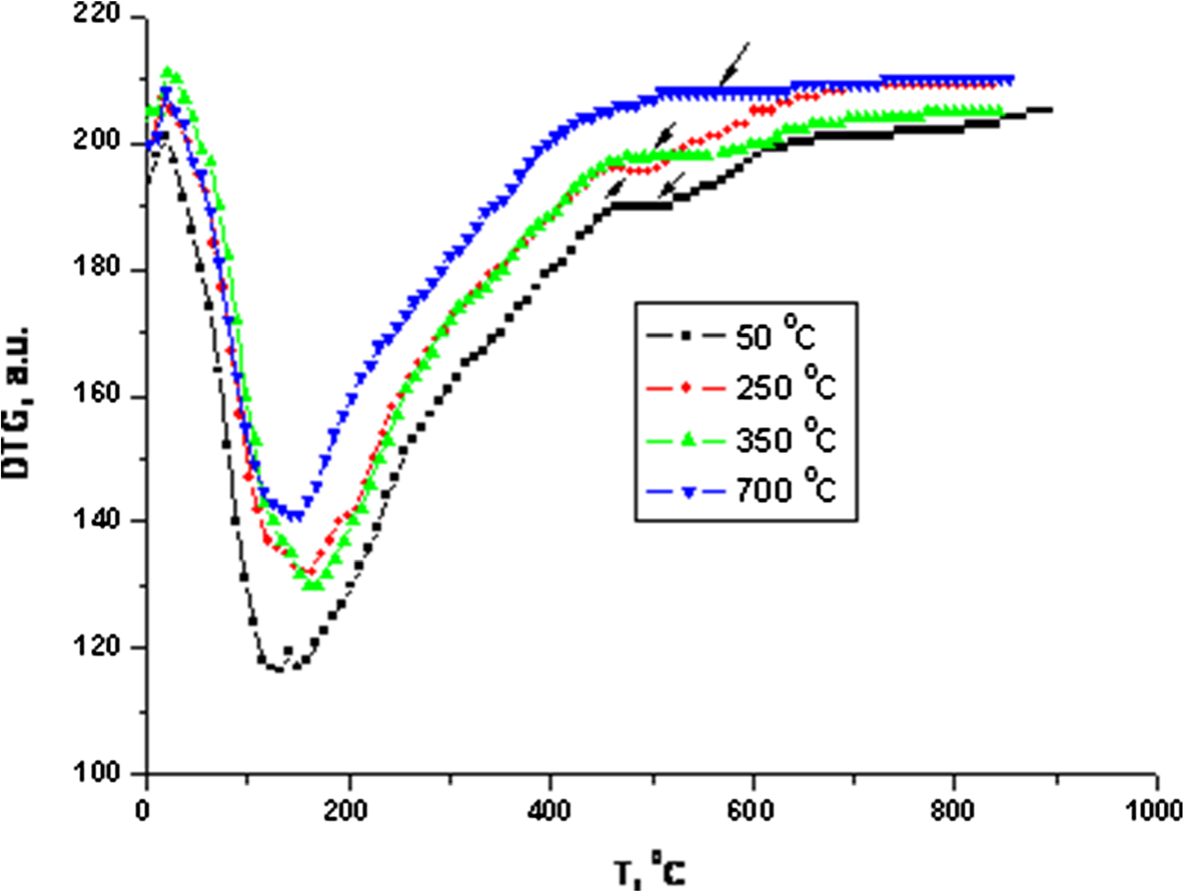
DTG-curves of Transcarpathian clinoptilolite previously heated at different temperatures

The results of our previous investigations [30] showed that efficiency of exchangeable cations and specific surface value of clinoptilolite change after thermal treatment of the sorbent. Similarly, specific surface area and sorption capacity of thermally treated clinoptilolite samples depend on the pretreatment temperature. Conseqently, an additional thermogravimetric experiment was carried out to investigate the thermal desorption of water from the clinoptilolite surface. Figure 2 illustrates TG-and DTG-curves of natural clinoptilolite and clinoptilolite thermally activated at 500 °C. It appears that TG-and DTG-curves have almost identical shapes. TG-curve entirely represents physical and chemical transformations of the sample during continuous heating. The observed plateau at 600–700 °С has an S-form which is typical for minerals. At the same time, TG-curve suggests the influence of previous thermal treatment of the sorbent on its adsorption capacity for water. These results could be confirmed by DTG-curves (Fig. 3) and data of the overall water loss at 900 °C for Transcarpathian clinoptilolite heated at different temperatures (Table 1). For instance, the samples thermally activated at 200–250 °C were characterized with a minimum of water loss (6.1 %). Relatively high amounts of water loss (9.4–9.8 %) were received for the clinoptilolite previously calcined at the temperature range of 300–500 °C. In this case, the reverse dependence between water loss amounts and temperature of thermal treatment was received. Nevertheless, previous heating of the sample at 700 °C again caused a sharp decrease in clinoptilolite water loss.

**Table 1:** Overall water loss at 900 °C for clinoptilolite samples previously heated at different temperatures

Temperature of the thermal pretreatment, °C	Water loss, %
Unheated form	10.4
50	10.4
100	10.0
200	6.1
200^а^	9.9
250	6.1
250^а^	9.9
300	9.8
350	9.7
500	9.4
700	6.6

^а^Clinoptilolite samples heated at the same conditions but additionally kept for 72 h in air with relative humidity of 70 %

Consequently, it could be proposed that, the temperature of the zeolite thermal pretreatment influences on a rate of adsorption equilibrium rather than on its sorption capacity of water at 200–250 °C. This suggestion could be confirmed by the fact that weight losses were 9.9 % for the sorbent previously heated at 200 and 250 °C and then kept for 72 h in air with relative humidity of 70 %.

These effects appeared from decelerated process of rehydration, since changes of clinoptilolite porous structure were still reversible at 200–250 °C. On the contrast, an irreversible deep amorphization of Transcarpathian clinoptilolite occured at 700 °C [33]. In general, DTG-curve is a differential curve of the sample weight change and describes the dependence of the weight loss rate from the temperature. For the studied clinoptilolite samples, DTG-curves were asymmetrical and some characteristic points were identified (Fig. 3). A broad DTG-maximum observed at low temperatures indicates the intensive thermal desorption of water. It should be noted that maximum rates of water loss were observed for all studied samples in the temperature range from 110 to 130 (140) °C, independently from previous thermal treatment conditions. Furthermore, this dissymmetry of the DTG-maximum could be caused by overlapping between a few quasielementary maximums, which suggests at least two types of molecular water physically bonded to the clinoptilolite surface. In this temperature range, the water loss did not significantly varied with the temperature of the sorbent preparation (6.3–7.0 %), except for the sample calcinated at 700 °C (4.4 %). An isokinetic region (indicated by arrows on Fig. 3) was observed on DTG-curves at the temperature of 440–560 °C for all samples, apart from the sorbent previously heated at 700 °C.

Moreover, an undefined DTG-maximum was found for the clinoptilolite previously calcined at 250 °C. It is known that a deep dehydroxylation of silica occurs in this temperature range [38], and practically all OH-groups appear to be isolated at temperature over 400 °C. Consequently, the fully dehydroxylated surface is covered with oxygen atoms because of recombination of two hydroxyl groups and release of water molecules. This process demands reorganization of the surface atoms and also should be activated. Since surface dehydroxylation of the Transcarpathian clinoptilolite considerably diminishes the amount of surface OH-groups responsible for the sorption of trace elements ions, the terbium sorption effectiveness of clinoptilolite heated above 400 °C was significantly decreased (Fig. 1).

It has been reported [39] that water molecules in the hydrated zeolite could form the cyclic hexamers with oxygen atoms of the sorbent framework. Due to these hydrogen bonds, water molecules did not contain free OH-groups. Moreover, such cyclic hexamers prevent sorption of large cationic aqua and hydroxo complexes of metal ions. Desorption of ligand water molecules caused a simultaneous destruction of hydrogen bonds and cyclic hexamers at the temperature above 200 °C. As a result, a number of free OH-groups considerably increased due to water molecules of the broken hexamer, which were still bonded to the zeolite framework. Since the surface OH-groups are mainly active sorption centers towards trace elements ions, a noticeable improvement of sorption properties of the clinoptilolite heated in the temperature range of 250–350 °C towards terbium ions (Fig. 1) was connected with the increased number of surface hydroxyl groups provided by water molecules and surface silanol (Si–OH) groups.

Permissible multiple contents (С_ion_/С_Tb(III)_) of ions common in waters and wastewaters, that do not change the maximum sorption capacity of clinoptilolite towards terbium ions, are led in Table 2. Increasing of the admixture concentration beyond a define value leads to the reduction in sorption effectiveness of the zeolite.

**Table 2:** Tolerance limits of some ions for terbium(III) sorption from aqueous solution of Transcarpathian clinoptilolite (concentration of Tb(III) – 1 μg∙mL^−1^; pH 8.25)

Species	Tolerance limit (C_ion_/C_Tb(III)_)
NH_4_^+^, NO_3_^−^	2000
Na^+^, K^+^	1500
Zn^2+^, Ca^2+^	50
Cl^−^	2500
Mg^2+^	300
SO_4_^2−^	1000

A 7 M solution of HNO_3_ and a 1 M solution of NaCl acidified with a hydrochloric acid to pH 2.5 were preferred as desorbents for terbium(III) ions, because each of them provided almost full recovery of lanthanide (Table 3). As a result, the developed solid phase extraction procedure could be applied for the spectrophotometric determination of trace amounts of terbium(III) ions in aqueous samples.

**Table 3:** Desorption effectiveness of terbium(III) ions from clinoptilolite^a^

Eluent	Desorption (%)
1 M NaCl	75
1 M NaCl (adjusted to pH 2.5 with HCl)	95–100
2.8 M HNO_3_	80
7 M HNO_3_	100
3.6 M H_2_SO_4_	30
2.4 M HCl	60
6 M HCl	70

^a^Flow rate of eluent through adsorption system = 1 mL∙min^−1^; volume of the eluent employed = 15 mL

### Sample preconcentration procedure

The sorbent was grounded in a ball mill to 0.20–0.31 mm size and washed with distilled water. After drying at room temperature, the clinoptilolite sample was heated in the oven at 350 °C for 2.5 h and then stored in a desiccator. 0.5–2 L water sample was acidified with nitric acid to a pH value approximately 1 and heated on a sand bath for 1 h. After the filtration through the dense paper filter, the pH of the water was adjusted to 7 with a sodium hydroxide solution, and then a borate buffer was added to maintain pH 8.25. The final concentration of a borate buffer in the sample solution was 2.5 · 10^−4^ М. The obtained solution passed through SPE cartridge filled with 0.6 g of the prepared sorbent at a flow rate of 3 mL · min^−1^. After the loading of the sample, the cartridge was washed with 50 mL of double-distilled water at the same flow rate. Whereas, terbium(III) ions were desorbed with 15 mL of a 1 M sodium chloride solution acidified with a hydrochloric acid to pH 2.5 at a flow rate 1 mL · min^−1^. 5 mL of double-distilled water was added to the eluate, and the pH was adjusted to 1 with hydrochloric acid. The obtained solution was made up to 25 mL volume with double-distilled water and thoroughly mixed. Terbium(III) content in the solution was determined spectrophotometrically with arsenazo III as an indicator. This procedure is described in detail in the experimental part. Overall, the proposed method for determination of Tb(III) ions had a linearity range from 2.5 to 200 ng · mL^−1^. The detection limit was found to be 0.75 ng · mL^−1^, and this parameter was calculated using the following equation:

$$ DL=3Sb/m, $$

where *Sb* is a standard deviation of blank and *m* is a slope of the calibration curve. The sample preparation method was tested on a model solution with the composition similar to natural water. As can be seen from Table 4, the components of waters do not have considerable influence on the determination of trace amounts of terbium(III) ions. The analyte recoveries from spiked tap water were above 93 %.

**Table 4:** Determination of terbium(III) ions in the synthetic water sample with the composition similar to natural surface waters after ions preconcentration with clinoptilolite (*n* = 3, *P* = 0.95)

Composition of synthetic solution, mg∙L^−1^	Volume of synthetic solution, mL	Enrichment factor^a^	Concentration of Tb(III), ng∙mL^−1^		Recovery, %	RSD, %
			Added	Found		
Na^+^(20), K^+^(5),	600	40	100	97 ± 3.9	97	1.6
Mg^2+^(1), Ca^2+^(10),	600	40	50	51 ± 2.6	102	2.08
Fe^3+^(0.5), SO_4_^2−^(5)	1050	70	10	9,8 ± 0.98	98	4.02
Cl^−^(20), HCO_3_^−^(50),	1950	130	5	4,7 ± 0.53	94	4.51
NH_4_^+^(1), Zn^2+^(0.005), NO_3_^−^(1)	1950	130	3	2,8 ± 0.49	93.3	7.1
	1950	130	0	N.D.^b^		

^a^Enrichment factor = volume of sample/volume of eluent

^b^N.D. < Detection limit

## Conclusions

A solid-phase extraction procedure was developed for spectrophotometric determination of trace amounts of terbium(III) ions in aqueous solution. Transcarpathian clinoptilolite heated at 350 °C for 2.5 h was applied as a SPE sorbent. Maximum sorption ability towards terbium(III) ions was observed after thermal activation of the clinoptilolite. The results of thermogravimetric investigations indicated a relationship between changes of zeolite structure during dehydration processes and its sorption abilities. It was shown that previous thermal treatment of clinoptilolite has an influence on its sorption capacity and the overall water loss, which suggests only partial reversibility of zeolite rehydration. Due to this fact, thermally activated samples of clinoptilolite maintain their sorption abilities towards trace elements ions, especially terbium(III) ions, in aqueous solutions.

The same sorption values were obtained for terbium(III) solutions with pH 8.25 regulated with either a borate buffer or sodium hydroxide. A buffer solution maintains a pH value, which permits to improve metrological characteristics of the measurements. Moreover, in this case the constant ionic strenght minimizes influence of other water components on the sorption process of Tb(III) ions. The developed method offers a possibility to concentrate trace amounts of terbium(III) ions in the presence of water components. Permissible multiple contents of competing ions for terbium(III) ions sorption were the following: 2500 (Cl^−^), 2000 (NH_4_^+^, NO_3_^−^), 1500 (Na^+^, K^+^), 1000 (SO_4_^2−^), 300 (Mg^2+^), 50 (Ca^2+^, Zn^2+^). A 1.0 M sodium chloride solution acidified with hydrochloric acid to pH 2.5 was used for quantitative desorption of terbium(III) ions., An enrichment factor of 130 was obtained under the optimum conditions. A wide range of linearity (2.5–200 ng · mL^−1^) with detection limit of 0.75 ng. mL^−1^ was achieved. The developed procedure was applied for the determination of terbium(III) ions in technological solutions, where recoveries and RSD values were 93.3–103.0 % and 1.6–7.1, respectively.
